# Web-Based Mind-Body Tactical Resilience Training Program for First Responders: Pre-Post Study Assessing Feasibility, Acceptability, and Usability

**DOI:** 10.2196/40145

**Published:** 2023-02-01

**Authors:** Leona Tan, Mark Deady, Olivia Mead, Rebecca M Foright, Eric M Brenneman, Jamie R Yeager, Richard A Bryant, Samuel B Harvey

**Affiliations:** 1 School of Psychiatry University of New South Wales Sydney Australia; 2 Black Dog Institute University of New South Wales Randwick Australia; 3 YogaShield Yoga For First Responders Castle Rock, CO United States; 4 Department of Anatomy and Cell Biology University of Kansas Medical Center Kansas City, KS United States; 5 School of Psychology University of New South Wales Sydney Australia

**Keywords:** resilience training, first responders, web-based intervention, mental health, mind-body, posttraumatic stress disorder, PTSD, prevention

## Abstract

**Background:**

First responders report elevated rates of mental disorders, including posttraumatic stress disorder (PTSD), yet many are reluctant to seek care. Preventative resilience training programs attempt to proactively address this issue, and there is evidence showing promise for programs targeting cognitive processes. However, these programs rarely address the physical health conditions associated with PTSD. There is emerging evidence of mind-body exercise training improving PTSD symptoms as well as its associated physical health symptoms. However, the feasibility and acceptability of delivering a web-based mind-body resilience training among first responders are not yet known.

**Objective:**

This study aimed to evaluate the feasibility, usability, and acceptability of a web-based mind-body tactical resilience training program designed for first responders. In addition, we explored the preliminary effectiveness of the training program on mental health outcomes, adaptive cognitive strategies, and work productivity.

**Methods:**

A total of 42 first responders based in the United States enrolled in the web-based training program. Participants were administered web-based surveys before enrolling in the 6-week web-based program and at the end of the program. The primary outcomes of feasibility were measured using the number of training hours, program adherence rates, and self-reported data on frequency of practice. Acceptability and usability were measured using self-reported data. Secondary outcomes were symptoms of PTSD, psychological distress, emotion regulation, stress mindset, psychological preparedness, and work performance.

**Results:**

Overall, the training program was feasible based on the median number of training hours spent on the web-based program (7.57 hours out of an expected total of 6 to 9 hours), and 55% (23/42) of the enrolled participants completed more than half of the program. Although acceptability, usability, and frequency of practice were rated as high, this was based on only 29% (12/42) of the respondents who provided follow-up data. Secondary outcomes showed a significant improvement in the adaptive cognitive strategy of the stress mindset, with a mean difference of –5.42 (SD 4.81; 95% CI −8.475 to −2.358; *t*_11_=−3.898; *P*=.002). All other secondary outcomes were not significant. However, the secondary outcomes were exploratory only, and this study was neither designed nor powered to adequately assess efficacy.

**Conclusions:**

These findings suggest that a mind-body tactical resilience training program delivered in a web-based format is feasible and acceptable among first responders; however, further refinements may be required to improve adherence rates. Further research using a larger, more rigorous trial design is warranted to examine the effectiveness of this type of training as a possible prevention or treatment strategy for this population.

## Introduction

### Background

First responders are regularly exposed to trauma and report elevated rates of mental health morbidities, including depression, suicidal thoughts and behavior, heavy alcohol use, and posttraumatic stress disorder (PTSD) [1-3]. Currently, PTSD remains a debilitating and challenging condition to treat. Although trauma-focused psychotherapies are a first-line treatment with a strong evidence base [4], many first responders are reluctant to engage in conventional mental health care when needed because of stigma and barriers to care [5,6]. In an effort to address these challenges, many first responder organizations are increasingly adopting mental health training programs as part of their workplace mental health promotion and prevention strategies. However, little is known about the effectiveness of these programs in preventing the development of mental disorders, in particular PTSD, because of the absence of high-quality studies in first responder populations [7,8].

A type of training rising in popularity in many first responder organizations is resilience training. This training seeks to prevent the development of psychiatric illnesses by equipping individuals with adaptive coping skills that would enable them to maintain psychological health despite exposure to adversity [9]. The concept of resilience has an obvious appeal to many high-risk organizations such as first responder agencies, where regular exposure to trauma is an unavoidable part of the role, and represents a promising strategy that could protect trauma-exposed individuals from psychological harm [9]. Current guidelines and best-practice frameworks on workplace mental health recommend improving employee resilience through preventative interventions as part of a broader strategy to develop mentally healthy workplaces [10]. Researchers have also recommended that resilience training interventions for first responders target risk factors that best predict the development of psychiatric disorders and that are potentially modifiable before the onset of the disorder [8]. Some of the modifiable risk factors that have been previously identified are behavioral disengagement and maladaptive cognitions such as cognitive bias and physical inactivity [11,12].

To date, promising evidence has been found for preventative resilience training targeting cognitive processes in military settings, another high-risk occupation group. A randomized controlled trial (RCT) on Israeli infantry soldiers examining the impact of a preventative attention bias modification training found that participants who received 4 sessions of attention bias modification training delivered before combat deployment had a significantly lower PTSD rate following combat exposure compared with those who did not receive the training. Another RCT on military cadet officers compared the effect of 2 other types of resilience training, one involving the use of a single session on coping skills using arousal training and adaptive thinking styles and the other involving multiple sessions of guided self-reflection that targeted maladaptive cognitions by reframing the experience of stress using more adaptive cognitive strategies such as growth and stress-is-enhancing mindsets [13]. The results showed long-term improvements in depression and anxiety symptoms as well as perceived stressor frequency among those who received the multisession self-reflection training compared with recipients of the single-session training on coping skills. Although further studies are needed to determine the generalizability of the findings to first responder populations, these studies show some promise for resilience training targeting cognitive processes. Although these findings show promise for improving cognitive processes and mental health outcomes, it is imperative that resilience training for first responders also addresses physical health symptoms associated with PTSD [11,12]. A recent meta-analysis found that trauma-exposed individuals were 2.7 times more likely to experience functional somatic syndrome, which was also strongly associated with PTSD [14]. Furthermore, first responders with PTSD are more likely to experience increased somatic symptom burden compared with those without PTSD [15], yet these symptoms are rarely addressed in mental health treatment or preventative strategies. Previous reviews on first responders and the military show promise for physical activity interventions in improving PTSD symptoms as well as physical health symptoms associated with PTSD [16,17]. These findings call for better integration of physical and mental health in preventative resilience training for first responders, which is especially pertinent for this population group that requires high levels of physical fitness to be maintained to perform work that is often physically demanding.

A type of physical activity intervention that uniquely integrates both psychological and physical health strategies is yoga training, a type of mind-body exercise that combines breathing techniques, controlled physical movement, and mindfulness practices simultaneously [18]. Previous reviews have found promising evidence of these interventions reducing PTSD symptoms [19-22]. Preliminary evidence has also been found for yoga in improving regulation of various physiological processes such as the autonomic nervous system, reducing inflammation, and improving symptoms of somatization disorders that are also associated with PTSD [23-25]. Although the evidence for yoga training has been limited by low-quality trials, current guidelines consider yoga as a potential priority candidate for further research on PTSD prevention and treatment [26]. There is also growing acceptability of this type of training among veterans and military personnel. In a large national sample of veterans in the United States, >50% of users of the Veterans Health Administration, the nation’s largest health care system, reported using a complementary and integrative approach for common physical and mental health conditions, where approximately 1 in 3 used mindfulness and 1 in 4 regularly engaged in yoga practices [27]. Moderate effect sizes have been found in veteran samples for the treatment of PTSD [20]. Favorable results were also found in an RCT of deployed military personnel, where yoga training was shown to be effective in improving state and trait anxiety as well as quality of life [28]. Given their apparent acceptability, good fit with culture, and emerging evidence for improving PTSD symptoms and its associated physical health symptoms, mind-body interventions may be a promising preventative resilience training strategy for first responders [4,29].

Indicative evidence has also been found for programs that include mind-body approaches using a web-based format [30,31]. A recent large-scale trial of a web-based behavioral activation and mindfulness application developed specifically for male-dominated occupation groups, which also included firefighters, found promising results for reducing depression and anxiety symptoms as well as improving work performance [30]. Another RCT on a web-based mindfulness-based resilience training targeting cognitive strategies was found to increase psychological resilience among firefighters [31]. Furthermore, a 2021 systematic review found that web-based delivery of yoga training was feasible based on attendance and rates of practice [32]. However, there was considerable heterogeneity across the studies, including how outcomes were measured. Although it is not known whether the feasibility of web-based delivery for this type of intervention may be applicable to first responders, there is preliminary evidence that a web-based mental health–informed physical activity intervention is feasible and acceptable based on a previous study on first responders and their partners [33]. Taken together, these recent developments suggest that further research is warranted to determine whether a web-based format of mind-body resilience training is feasible and acceptable for active-duty first responders.

### Objectives

A novel web-based mind-body tactical resilience training program was developed specifically for first responders to address these research gaps. The primary aim of this study was to ascertain the feasibility, acceptability, and usability of a web-based mind-body tactical resilience training program that is culturally informed and job-specific to first responders. In addition, the study explored the secondary outcomes of the preliminary effectiveness of the web-based training program in (1) reducing symptoms of PTSD and psychological distress; (2) modifying mindsets about stress, emotion regulation, and perceived psychological preparedness; and (3) improving work productivity.

## Methods

### Development of the Intervention

**Figure 1:**
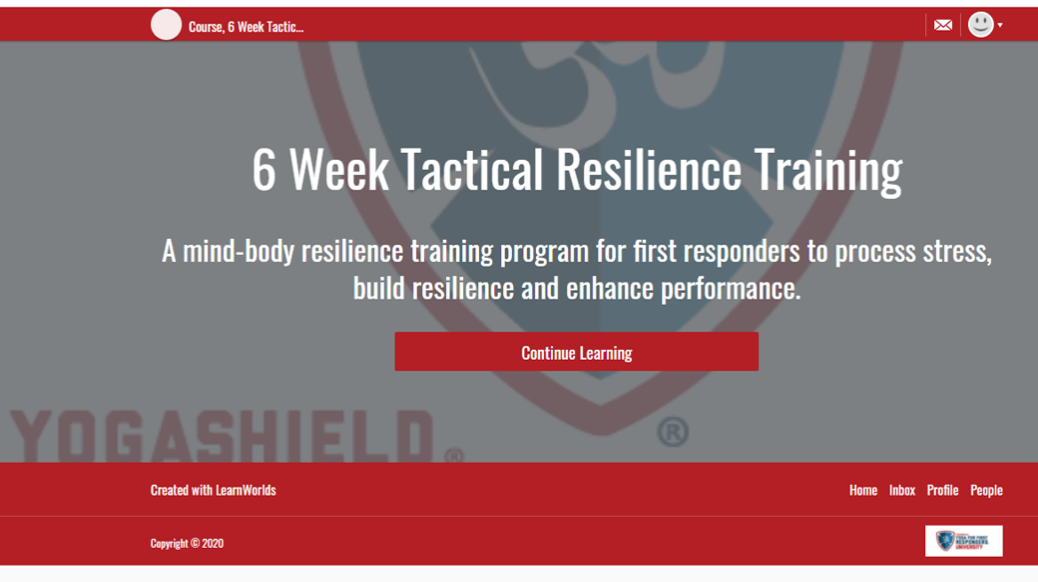
Screenshot of the Online 6-Week Tactical Resilience Training program landing page.

**Figure 2:**
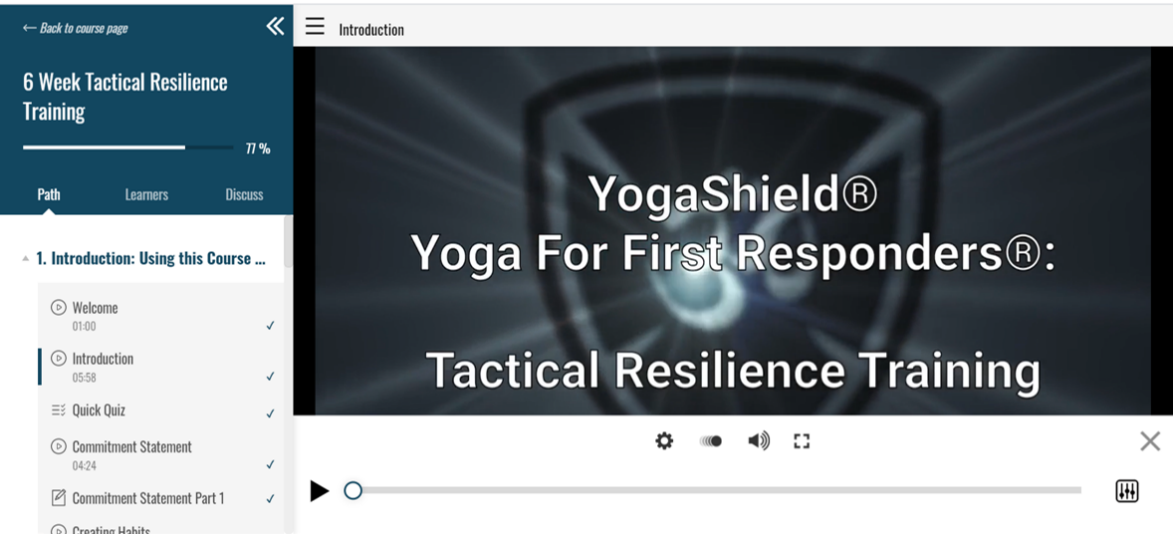
Screenshot of the Online 6-Week Tactical Resilience Training program dashboard.

**Figure 3:**
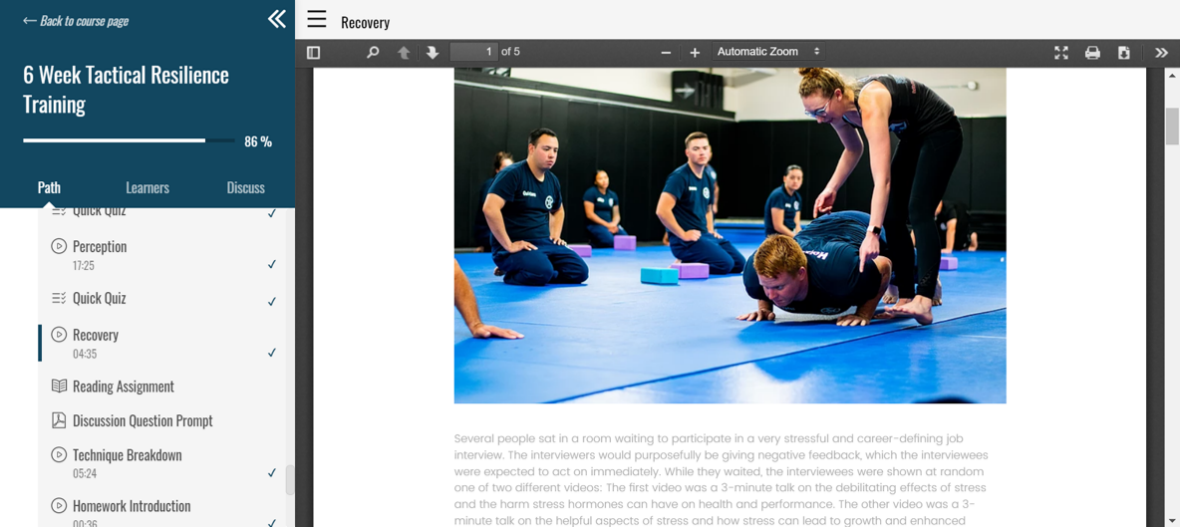
Screenshot of an article.

**Figure 4:**
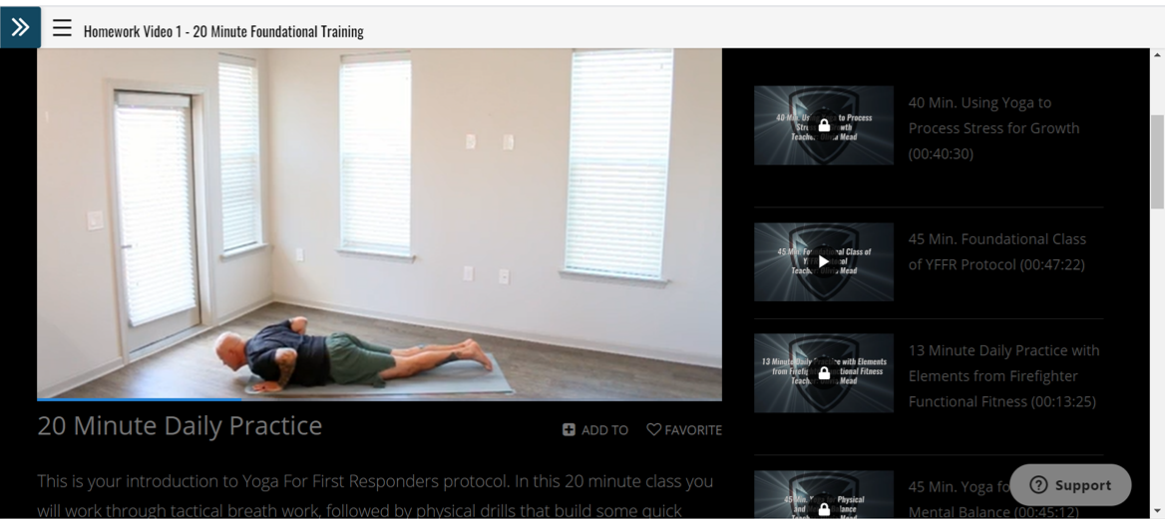
Screenshot of a homework video.

The Online 6-Week Tactical Resilience Training program (Figures 1 to 4) is a novel web-based mind-body tactical resilience training program for first responders that was developed by YogaShield Yoga For First Responders (YFFR), a registered nonprofit organization based in the United States. The development of the program involved modifying a preexisting YFFR in-person training protocol into a web-based format in accordance with guidelines on pilot and feasibility trials as well as a previous review on web-based lifestyle interventions [34,35]. To maximize web-based engagement, the program adopted recommended strategies from a previous review on web-based prevention for lifestyle behaviors [35], which included a modular setup, a <10-week program, interactive components with facilitators and peers, and persuasive technology features such as positive feedback and extrinsic motivation by rewarding completers with a certificate of achievement. The aim of the program was to provide first responders with practical self-regulation and cognitive skills to process stress, build resilience, and enhance work performance using mind-body techniques in language and exercises that were applicable to the work of a first responder.

### Consultation

As part of the modeling phase, first responders based in the United States were previously surveyed on the relevance of techniques and sections as well as web-based content preferences to determine the feasibility, acceptability, and usability. This consultation phase was not part of the research study and was conducted by the YFFR program developers before the development of the web-based program. To consult with first responders, YFFR placed an advertisement in their regular web-based newsletter to their local first responder network asking for feedback on the development of a web-based training program. Interested first responders were directed to a web-based survey as part of the advertisement. A total of 78 first responders answered this anonymous web-based survey, half of whom (39/78, 50%) had attended a previous YFFR training. Questions were asked on general interest in a web-based mind-body resilience training program; current yoga and meditation practices (if any), including frequency and place of practice; preferences for a web-based program, including time commitment and preferred time of day; helpful reminder cues; learning tools; use of technology; preferences for live classes; and strategies to encourage training participation. Examples of the questions include the following: *If you were participating in an online yoga training program, which learning tools would you prefer?* *When using an online training program, how many days a week would you be able to commit?* *When using an online training program how much time per day could you commit?* *Would you be interested in participating in this online training program when it is launched?* Adjustments were made to the training content based on the feedback received wherever appropriate.

### The Web-Based 6-Week Tactical Resilience Training Content

The web-based program was based on the YFFR in-person training protocol, which was designed to provide proactive resilience training specifically for first responders. It is primarily based on the principles of traditional Hatha yoga, which involves regulation of breathing using synchronized movements and postures, as well as trauma-sensitive yoga, a trauma-informed approach designed with trauma survivors in mind to cultivate a safe environment [36]. These yoga principles were applied to the context of first responder work and culture to make this type of training accessible as a form of workplace training program. The web-based program expanded on the in-person training protocol by providing background educational material in addition to demonstrations of mind-body techniques. It consisted of 6 modules primarily based on habit formation for behavior change, psychophysiology of the stress response, and cognitive reframing and mind-body skill training. Specifically, the six modules were (1) developing sustainable habits; (2) foundations of YFFR; (3) tactical breathwork and yoga techniques; (4) processing stress and self-regulation strategies; (5) building mental, physical, and emotional resilience; and (6) enhancing job performance from decision-making to tactical skills. Table 1 provides an overview of each module. Each section had to be completed sequentially and contained a series of short lecture and technique breakdown videos (ranging from 5 to 10 minutes each), brief articles and quizzes, and guided homework video or audio recordings of yoga and mindfulness techniques ranging from 5 to 30 minutes. Each module took approximately 1 to 1.5 hours to complete. The homework videos could be accessed directly through the web-based program as well as through a separate app, which participants were given access to upon registration. The web-based program also featured a discussion forum to allow for interaction between a trained instructor and participants. Participants also had the option of attending live group Zoom (Zoom Video Communications) classes with a YFFR instructor at a predetermined time. The first module was an introduction to the course with instructions on how best to use the course and how to integrate it into participants’ daily lives as a regular habit. Module 2 described the concepts of yoga and mindfulness and how the YFFR training protocol was specially designed to apply these concepts to the life and work of a first responder. Modules 3 to 5 described how yoga and mindfulness could be used as a tool to process stress, build resilience, and enhance the work performance of first responders. These modules also provided instructions and demonstrations of specific techniques such as breathwork and yoga sequences, which were also referred to as physical drills; cognitive declarations to help reframe perceptions of stress; and mindfulness practices. Instructions were also provided on how to apply these techniques during and after the activation of the stress response. Finally, module 6 concluded the overall training and provided additional resources and web-based community networks for participants to continue their training should they wish to do so.

Although the web-based training program was intended to be self-paced, participants were expected to complete the course within a 6-week period, with automated weekly reminders sent to prompt program adherence. The 6-week time frame was based on the expected time commitment of 1 week per module; however, participants were given an additional 2 weeks to catch up on any materials missed. The program was mobile-responsive, which made access easy using a desktop, laptop, tablet, or mobile phone.

**Table 1:** Course outline of the 6-Week Tactical Resilience Training program.

Module number	Module name	Description	Homework video or audio
1	Introduction: using this course	Introduction and habit creation	20-minute yoga sequence
2	Foundations of Yoga For First Responders	What is yoga, mindfulness, the missing skillset, and Yoga For First Responders	20-minute basic yoga practice 15-minute mindfulness
3	Processing stress	Processing stress, interoception and proprioception, and removing the armor—functional mobility training	15-minute AMRAP^a^ sequence for building heat 30-minute removing the armor
4	Building resilience	Resilience, neural pathways, perception, and recovery	16-minute sleep recovery 10-minute breathwork and mental reframing technique 1 10-minute breathwork and mental reframing technique 2
5	Enhancing performance	Flow, job- or life-specific application, and handling challenges	13-minute yoga sequence 13-minute yoga warm-up for SWAT^b^ operators
6	Conclusion: continuing your training	Additional web-based resources, community networks, final quiz, and certificate of completion	20-minute daily yoga practice 5-minute before-shift practice 5-minute after-shift practice

^a^AMRAP: as many rounds as possible.

^b^SWAT: special weapons and tactics.

### Study Design and Population

This study aimed to examine the feasibility, acceptability, and usability of the 6-Week Tactical Resilience Training program and explore the preliminary effectiveness of a web-based mind-body tactical resilience training program specifically designed for the needs of active-duty first responders. The study was conducted with a small group of first responders where all participants were allocated to receive the intervention. Participants comprised first responders located throughout the United States with no previous training or relationship with YogaShield YFFR.

### Recruitment

Participants were recruited through email advertisements that were circulated to YogaShield’s first responder networks located across metropolitan and regional areas throughout the United States. Using snowball sampling methods, these networks then distributed the email advertisements to their social and professional networks. First responders with no previous YFFR training were invited to participate in the study. Participants met the inclusion criteria if they were current active-duty frontline first responders, specifically firefighters, police, and paramedics, and dispatchers who were either in paid employment or in a volunteer capacity. Participants also had to currently reside in the United States, be aged ≥18 years, and speak English. The recruitment advertisement described the purpose of the study and what would be involved and contained a link to the study website as well as the participant information sheet and consent form. The advertisement also emphasized that participation in the study was voluntary and not a requirement of their job. Study recruitment occurred in February 2021 for approximately 3 weeks.

### Ethics Approval

Each participant completed their own informed consent form by indicating their agreement with a declaration statement. By agreeing to take part in the study, participants declared that they had read and understood the participant information sheet, could ask any questions they may have, understood the risks and benefits, and knew that they were free to withdraw at any point throughout the duration of the study without penalty or consequences. Agreement was expressed by checking a box and providing their name and email address. Participants were also informed that the study data would be deidentified before analysis. Compensation was not provided for participation. Ethics approval was obtained from the Human Research Ethics Committee and the University of New South Wales before the commencement of the study (HC200707).

### Procedure

Eligible participants who consented to the study were directed to complete the baseline assessment on the web. Upon completion, participants were provided with a hyperlink to register for the web-based training program by entering their name and preferred email address. Log-in credentials were automatically sent to participants’ designated email address. The web-based training program was made available at the end of the recruitment period on February 22, 2021, after which all the registered participants were able to commence the course. The web-based program was designed to be completed within a 6-week period, with an additional 2-week “catch-up” period. Although participants were encouraged to complete the program within 8 weeks, the program was still available for those who were unable to complete the web-based course within that time frame. Automatic email reminders were sent before the start of the web-based program, as were weekly reminders to continue working through the program. Web-based follow-up surveys were distributed upon completion of the web-based training program or at the end of the 8-week study period for those who had not yet completed the program within that time frame.

### Data Collection

The baseline and postintervention surveys were collected electronically via the REDCap (Research Electronic Data Capture; Vanderbilt University) platform [37,38] hosted at the University of New South Wales, whereas the number of training hours and program adherence statistics were obtained from the hosting platform of the Online Tactical Resilience Training program. In cases of nonresponse to the surveys, 2 automatic reminders were sent over the subsequent 14 days via the REDCap platform.

### Measures

#### Overview

Demographic information, including age, gender, occupation type, employment status, length of service, previous yoga or resilience training, and help seeking, was collected at the start of the baseline survey. Participants were also asked to indicate the approximate number of critical incidents that they had attended since becoming a first responder. The first 2 items on serious injuries and fatalities were based on a previous study on the impact of cumulative exposure on first responder mental health [1]. A total of 2 other items asked participants about any direct or indirect experience of physical or sexual assault or learning that any of these events had occurred to a close family member or friend. These questions were derived from Criterion A of the Diagnostic and Statistical Manual of Mental Disorders, Fifth Edition, PTSD diagnostic criteria [39].

#### Primary Outcomes

##### Feasibility

The feasibility of the web-based program was based on the number of hours spent on the program as well as program adherence data obtained from the hosting platform. On the basis of a previous feasibility study for web-based delivery of a similar mindfulness-based training with active-duty first responders, we established at least 50% of participants completing more than half of the program as the cutoff benchmark for feasibility [40]. To obtain insights into noncompletion, participants were asked in the web-based follow-up assessment to provide reasons for noncompletion using free text if they did not manage to complete the course in time. To examine the feasibility of the program for behavior change, participants were also asked in the follow-up assessment to indicate the frequency with which they had practiced the breathwork techniques, physical drills or yoga sequences, cognitive declarations, and meditation upon completion of the training program.

##### Usability and Acceptability

The web-based follow-up assessment also contained measures of usability and acceptability, which were adapted from a previous study on a web-based mental health training program for first responders and construction workers [41]. Usability was measured by asking participants to rate their level of agreement with the following statement: “It was easy to find the information I needed.” Responses were rated on a 5-point Likert scale ranging from *strongly disagree* to *strongly agree*. In addition, participants were asked to rate how useful they found key components of the course. This included the 4 main modules (Foundations of Yoga and Yoga For First Responders, Processing Stress, Building Resilience, and Enhancing Job Performance) as well as the type of learning materials, including video or audio lecture recordings, interactive exercises, prerecorded yoga videos, and live Zoom classes (if attended). Participants were asked to indicate the degree of usefulness using a 5-point Likert rating scale ranging from *not at all useful* to *very useful*. Items rated as useful or very useful were indicative of usability. Acceptability was measured using a 5-item scale adapted from the same previous study [41] to access their overall satisfaction with the web-based program. Participants were asked to indicate their level of agreement with the following statements: (1) the web-based course was engaging and interesting, (2) the program met their expectations, (3) the web-based course was useful, and (4) they believed that it helped them improve their overall well-being. Responses were rated on a 5-point Likert scale ranging from *strongly disagree* to *strongly agree*. Items that were rated as *agree* or *strongly agree* were indicative of acceptability. In addition, participants were asked if they would recommend the training program to their coworkers. Upon completion of the final module, participants were also given the opportunity to provide optional qualitative feedback on the overall course using a free-text typed response through a separate and anonymous Google Form that was embedded in the web-based program.

#### Secondary Outcomes

##### Posttraumatic Stress

The preliminary effectiveness of the secondary outcomes was assessed at baseline and at the 8-week follow-up time point. Symptoms of PTSD were measured using the PTSD-8 [42]. The PTSD-8 is an abbreviated version of the Harvard Trauma Questionnaire [43] and comprises three symptom clusters of the Diagnostic and Statistical Manual of Mental Disorders, Fourth Edition, criteria for PTSD: (1) intrusion, which comprises 4 items; (2) avoidance, which comprises 2 items; and (3) hypervigilance, which also comprises 2 items. Responses were rated on a 4-point Likert scale (1=*not at all* to 4=*most of the time*). The cutoff criterion for likely PTSD was established through a combination of at least one symptom with an item score of ≥3 from each of the 3 PTSD symptom clusters. The summed score provides the score for symptom severity. Good internal consistencies, as measured by the Cronbach α, were achieved in previous trauma-exposed samples of accident, sexual assault, and disaster survivors (Cronbach α=.83, .84, and .85, respectively) [42].

##### Psychological Distress

Psychological distress was measured using the 6-item Kessler Psychological Distress Scale (K6) [44]. The K6 is a brief screening tool for serious mental illness that detects mood or anxiety disorders and has high validity and reliability [44,45]. Participants were asked to indicate the frequency with which they had experienced a symptom in the previous 4 weeks using a 5-point scale (0=*none of the time* to 4=*all of the time*) [46]. Participants who scored ≥13 were classified as having a probable serious mental illness [45,47].

##### Emotion Regulation

Emotion regulation was assessed using the Emotion Regulation Questionnaire, a 10-item scale designed to measure respondents’ tendency to regulate their emotions through cognitive reappraisal or expressive suppression [48]. The cognitive reappraisal subscale comprises 6 items, whereas the expressive suppression scale comprises 4 items. Responses were rated on a 7-point Likert-type scale (1=*strongly disagree* to 7=*strongly agree*). Internal consistencies for both the cognitive reappraisal (Cronbach α=.89-.90) and expressive suppression (Cronbach α=.76-.80) subscales were high in various community samples [49].

##### Stress Mindsets

Mindsets on stress were measured using the Stress Mindset Measure [50], which assesses the extent to which an individual adopts a mindset according to which the effects of stress are either enhancing or debilitating. The scale consists of 8 statements regarding the effects of stress. Responses are rated on a 5-point scale (0=*strongly disagree* to 4=*strongly agree*). Negatively worded statements are reverse scored. Higher scores represent a stress-is-enhancing mindset. Internal consistency was high, with a Cronbach α of .87 [50].

##### Psychological Preparedness

Psychological preparedness was measured using a modified version of a previously validated subscale of psychological preparedness for natural disasters [51] and adapting it specifically to first responders. The previously validated subscale of Anticipation, Awareness, and Management was adapted to measure how psychologically prepared first responders felt in response to critical incident situations. The modified scale for the study comprised 7 items, and participants rated their responses using a 4-point scale (1=*not true of me* to 4=*exactly true of me*).

##### Work Performance

Work performance was assessed using a single item from the Health and Work Performance Questionnaire and 2 additional items pertaining to past-month sickness absence [52]. For work performance, participants were asked to rate their overall job performance for the previous 4 weeks using a scale from 1 to 10 (1=*Worst performance* to 10=*Top performance*). For sickness absence, participants were asked to indicate how many sick days they had taken in the previous 4 weeks and, if so, how many of these were because of mental health or emotional problems.

### Statistical Analysis

Descriptive statistics were used to demonstrate participants’ responses to the feasibility, acceptability, and usability of the Online Tactical Resilience Training program. Differences in the mean scores for each of the outcomes between baseline and the 8-week follow-up point were assessed using paired-sample 2-tailed *t* tests. The outcomes considered included symptoms of PTSD and psychological distress, emotion regulation, stress mindset, psychological preparedness, help seeking, and work performance. All statistical analyses were conducted using SPSS (version 26; IBM Corp) [53].

## Results

### Demographics

**Figure 5:**
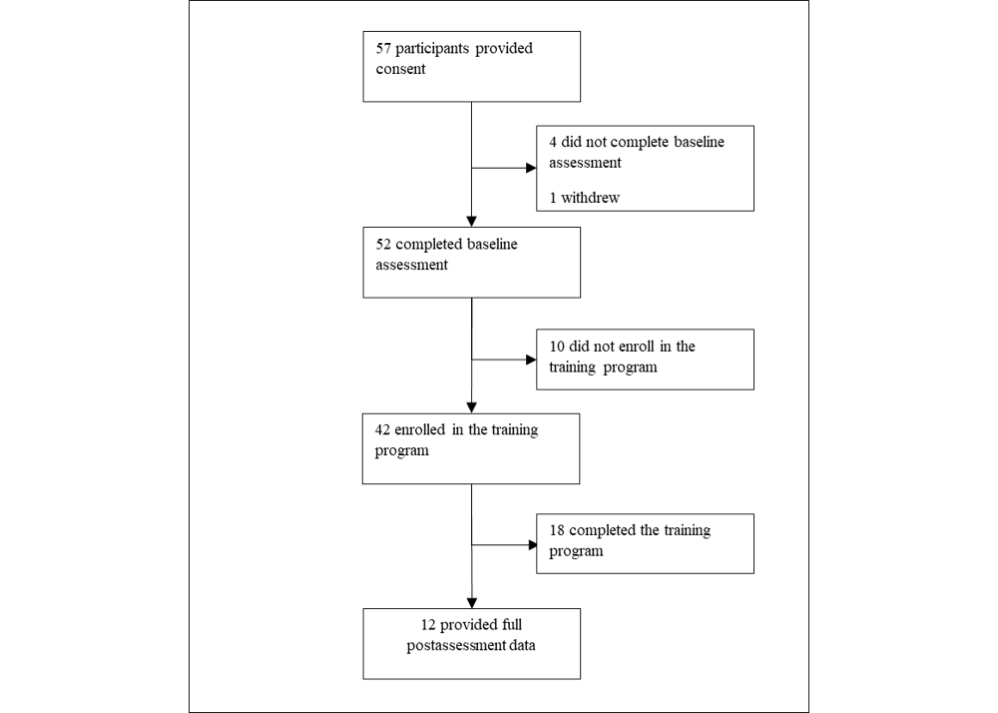
The Online 6-Week Tactical Resilience Training program feasibility study procedure flow.

A total of 57 participants consented to the study, of whom 52 (91%) completed the baseline survey. All 52 participants were invited to register for the web-based training program, of whom 42 (81%) registered and enrolled. Figure 5 shows the study procedure flow. Baseline characteristics of the study sample are outlined in Table 2. Of the enrolled participants, 29% (12/42) completed the follow-up assessment. No significant differences were found in age (*P*=.32), gender (*P*=.55), baseline PTSD (*P*=.28), or K6 cutoff scores (*P*=.61) between those who provided follow-up data and those who did not.

**Table 2:** Sample demographic characteristics (N=42).

Characteristics		Values
Age (years), mean (SD)		42.98 (7.61)
**Occupation group^a^, n (%)**		
	Law enforcement	17 (40)
	Firefighter or rescue worker	17 (40)
	Paramedic or emergency medical technician	13 (31)
	Dispatcher	3 (7)
**Employment status, n (%)**		
	Full time	41 (98)
	Part time	1 (2)
	Casual or seasonal or reserve	0 (0)
	Volunteer	0 (0)
**Gender, n (%)**		
	Man	25 (60)
	Woman	17 (40)
**Years of experience, n (%)**		
	1 to 5	3 (7)
	>5 to 10	4 (10)
	>10 to 15	4 (10)
	>15 to 20	14 (33)
	>20	17 (40)
**Previous yoga or resilience training, n (%)**		
	No	28 (67)
	Yes	14 (33)
**Number of serious injury critical incidents attended, n (%)**		
	1 to 5	1 (2)
	6 to 10	3 (7)
	11 to 20	4 (10)
	>20	28 (67)
**Number of fatalities attended, n (%)**		
	1 to 5	3 (7)
	6 to 10	5 (12)
	11 to 20	6 (14)
	>20	28 (67)
**Directly or indirectly experienced physical or sexual assault (number of times), n (%)**		
	0	9 (21)
	1 to 5	15 (36)
	6 to 10	7 (17)
	11 to 20	3 (7)
	>20	8 (19)
**Learned that any of the aforementioned events occurred to a close family member or friend (number of times), n (%)**		
	0	4 (10)
	1 to 5	29 (69)
	6 to 10	8 (19)
	11 to 20	0 (0)
	>20	1 (2)
**Modules completed**		
	0, n (%)	8 (19)
	1, n (%)	4 (10)
	2, n (%)	3 (7)
	3, n (%)	4 (10)
	4, n (%)	2 (5)
	5, n (%)	3 (7)
	6, n (%)	18 (43)
	Value, mean (SD)	3.64 (2.47)

^a^Occupation groups are not mutually exclusive.

### Feasibility

Analysis examining baseline predictors of program completion found no evidence that age (*P*=.09), gender (*P*=.41), baseline PTSD (*P*=.59), or K6 cutoff scores (*P*=.89) were able to predict which participants were more likely to complete the program. The total number of hours spent on the entire program varied considerably, with a median of 7.57 hours. The median number of hours spent on the web-based modules was 5.45, and it was 1.35 for the homework yoga videos, which could be accessed either via the web-based program or via a separate app. App use was not tracked as part of this study. Less than half (18/42, 43%) of the participants who enrolled in the tactical Resilience Training program completed all 6 modules of the course. More than half (23/42, 55%) completed >50% of the program, meeting the benchmark for feasibility. Half (21/42, 50%) of the enrolled participants completed >80% of the program. Of the 12 participants who completed the follow-up assessment, 10 (83%) completed the web-based program, whereas 2 (17%) did not—of these 2 noncompleters, 1 (50%) completed only 2 modules and the remaining participant (50%) completed 4 modules. Both respondents cited time constraints as the reason for noncompletion, with one of the respondents adding the program not being user-friendly as another reason for noncompletion. Respondents to the follow-up assessment indicated a high frequency of practice, with all respondents practicing the breathwork techniques at least 3 to 4 times weekly, most (11/12, 92%) practicing yoga sequences at least once or twice weekly, and 83% (10/12) practicing meditation and cognitive declaration techniques at least once or twice a week.

### Usability and Acceptability

Most respondents (10/12, 83%) agreed that it was easy to find the information they needed and that the main modules of the course were useful. Although most also rated the lecture videos (10/12, 83%), interactive exercises (11/12, 92%), and yoga videos (12/12, 100%) as useful or very useful, some respondents did not find the discussion forums (3/12, 25%) or live Zoom classes (if attended; 1/6, 17%) useful.

Acceptability was high among those who responded to the follow-up survey, with most respondents (11/12, 92%) agreeing that the course was engaging and interesting and met their expectations and that they would recommend the program to their coworkers. All respondents (12/12, 100%) rated the overall course as useful and believed that it helped improve their overall well-being.

The qualitative feedback received on what participants learned from the training was primarily positive. Some examples of comments included the following: “It felt good to do them, I learned something new,” “I felt so much better and the days I didn’t do my yoga I felt not so focused,” and “I learned that it is okay to put myself first, and that I can control my mindset.” The only major negative feedback regarding the course was the length of time required to complete it, as described by a participant: “Course material was more than expected and took more time, this not allowing time for homework.”

### Secondary Outcomes

Following their use of the web-based Tactical Resilience Training program, participants reported significantly higher stress-is-enhancing mindsets compared with the baseline, with a mean difference of –5.42 (SD 4.81; 95% CI −8.475 to −2.358; *t*_11_=−3.898; *P*=.002). Total PTSD symptoms showed a nonsignificant trend toward reduction, with a mean difference of 1.58 (SD 2.50; 95% CI −0.007 to 3.174; *t*_11_=2.191; *P*=.05). All other secondary outcomes were not significant. Table 3 displays the baseline and posttraining scores for the secondary outcomes.

**Table 3:** Baseline and posttraining score mean and SD for secondary outcome variables (N=12).

Measure	Baseline score, mean (SD)	Posttraining score, mean (SD)	*P* value
PTSD-8^a^	21.67 (5.23)	20.08 (6.19)	.05
K6^b^	7.33 (5.53)	6.17 (6.19)	.53
SMM^c^	15.33 (5.30)	20.75 (7.38)	.002
ERQ^d^ (cognitive reappraisal)	30.42 (5.87)	34.42 (4.66)	.11
ERQ (expressive suppression)	15.08 (5.09)	15.33 (5.91)	.86
Psychological preparedness	22.18 (4.12)	23.91 (3.11)	.19
Performance	7.45 (0.93)	7.73 (1.27)	.34
Sickness absence	3.00 (1.41)	1.50 (0.71)	.21

^a^PTSD-8: 8-item abbreviation of the Harvard Trauma Questionnaire.

^b^K6: 6-item Kessler Psychological Distress Scale.

^c^SMM: Stress Mindset Measure.

^d^ERQ: Emotion Regulation Questionnaire.

## Discussion

### Principal Findings

The primary aim of this study was to examine the feasibility, acceptability, and usability of a web-based mind-body tactical resilience training program that was culturally informed and specific to first responders. Results from the number of hours spent and program adherence rates suggest that the web-based mind-body resilience training program is feasible among active-duty first responders. Although acceptability, usability, and frequency of practice were rated as high, this was based on only 29% (12/42) of the respondents who provided follow-up data. Analyses of secondary outcomes showed a significant improvement in mindsets on stress, whereas no other significant changes were found for any of the other secondary outcomes. However, the findings on secondary outcomes were exploratory only, and our study was underpowered to assess effectiveness. To the best of our knowledge, this is the first time that a web-based mind-body tactical resilience training program designed specifically for first responders has been developed and tested.

The total number of training hours provided an indication of feasibility; however, this varied considerably between participants, with a median of 7.57 hours for the overall web-based program. A median of 5.45 hours was spent on the web-based modules, whereas a median of 1.35 hours was spent on the homework yoga videos. However, homework videos were only tracked via access through the web-based program. Access via the separate app was not tracked as part of this study as this was only intended to supplement participants’ learning and homework videos could only be accessed upon completion of the accompanying module. It is possible that the actual use of the homework videos was higher as a result. Program adherence for the web-based delivery of the mind-body resilience training surpassed the benchmark based on a previous mindfulness-based feasibility study on first responders [40], with half (21/42, 50%) of the enrolled participants in this study completing >80% of the program. Furthermore, our completion rate of 43% (18/42) is consistent with a previous study on frontline health care workers that examined the feasibility of a web-based yoga program of similar duration [54]. Although these findings indicate comparable feasibility of this web-based program, our study was not exempt from the issue of retention, which is an ongoing challenge for many web-based psychological interventions. Previous studies on these interventions have found varying dropout rates, ranging from 2% to 83% [55-57]. Dropout rates of approximately 50% are also a common finding for existing PTSD treatments in populations with high posttraumatic stress [58]. Although the reasons for noncompletion of this web-based mind-body resilience training program are not clear, there may be several reasons for this that can be inferred from our study. First, the time required to complete the program may have been too long, as suggested by 1 respondent. The entire program was expected to take approximately 6 to 9 hours to complete, and it is possible that a brief or more condensed version of the existing program would have encouraged better completion rates. A previous feasibility trial for web-based yoga training for posttraumatic stress found that most participants did not meet the set benchmark of 60 minutes per week despite initially reporting this benchmark as appropriate [59]. Further research on the optimal length and frequency of use for this type of program is required to improve feasibility in this population.

Second, the web-based self-guided format for this type of program may not have met the needs of some first responders who may have preferred the presence of an instructor. Previous reviews have demonstrated the superiority of guided web-based interventions to unguided web-based interventions in improving completion rates and symptom reduction, likely because of the provision of human support, feedback, and encouragement in completing the intervention and applying newly learned skills in practice [60,61]. It is also unknown if a group format may yield better results than the individual format of the existing program. A previous study on a mental health–informed physical activity intervention using a private Facebook group showed high retention rates among first responders and their partners [33]. The additional advantage of social media that participants were already engaging with may have also contributed to these rates. Although the inclusion of live Zoom classes attempted to address these issues, there was difficulty in scheduling classes that could accommodate all participants because of the varying shift patterns of first responders. Future iterations of this web-based mind-body resilience training program may benefit from an instructor-led or group format as well as the use of social media.

Our study also experienced low retention rates in the follow-up assessment. Although it is not known why this high loss occurred, this may have been due to several factors. First, it is possible that the follow-up assessment, which comprised multiple questionnaires, was too lengthy. The lack of incentives to complete the assessments may have also contributed to this high loss. Furthermore, as the research team was based overseas, there was little opportunity to communicate the benefits of the research program other than via email. Improving follow-up procedures and expanding methods of contact such as the use of social media and better recruitment procedures through first responder agencies may help improve retention rates in future studies. Finally, it is also possible that retention rates for the follow-up assessment and program completion were affected by the COVID-19 pandemic, an unprecedented time in history and a particularly demanding one for first responders because of the nature of their roles. The study trial occurred in the middle of the COVID-19 pandemic, and although it is not known if retention was directly affected by the uniqueness of the time, it is possible and understandable that adherence to the follow-up assessment and web-based program was not a priority for many first responders during this disruptive time of ongoing uncertainty.

Although our study was underpowered to detect measures of effectiveness, our significant finding on the stress mindset shows that it may be possible for this type of program to modify important perceptions of stress. Evidence is emerging on the influence of stress mindsets on perceived stress, physiological health, and mental health outcomes. Specifically, adopting a stress-is-enhancing mindset has been found to mitigate the development of depression and anxiety symptoms among college students with high levels of stress [62] and was found to moderate the effect of perceived and pregnancy stress on prenatal anxiety and depression as well as ratings of pregnancy as a stressor on postpartum depression [63]. Furthermore, an RCT of resilience training targeting cognitive evaluation skills in adults aged >50 years determined that adopting a stress-is-enhancing mindset, perceived stressor benefit, and coping efficacy were likely mechanisms for change in positive mental health and emotional outcomes [64]. However, as the primary goal of our study was to assess the feasibility of the web-based program, further research using a larger-scale controlled trial is required to determine the efficacy of such a program in modifying stress mindsets and their relationship to mental health outcomes.

Our work adds to the limited studies on high-risk occupations [12] and presents occupation-specific mind-body resilience training as a worthy consideration for web-based preventative resilience interventions for first responders. Our findings lend support to previous studies with first responders regarding the fact that web-based physical activity and mindfulness training is a feasible and acceptable form of resilience training for this occupation group [33,40]. Furthermore, the acceptable use of mind-body exercise to integrate both psychological and physical health strategies is consistent with growing acceptability and popularity as well as high satisfaction rates among veterans, another high-risk occupation group [65,66]. This novel web-based program also has numerous practical benefits, including the potential to bypass current mental health care barriers. Previous research has shown that stigma and structural issues such as cost and time are key barriers to help seeking for many first responders [67]; however, these could be overcome through work recovery strategies, mindfulness, and the use of technology [68]. This program attempts to address these barriers through 2 key approaches. First, the program is customized specifically for first responder work and culture and prescribes to their preferences for fitness and well-being programs [69]. All accompanying yoga, mindfulness, and meditation demonstrations were specifically adapted to show how such techniques could be applied to the life of a first responder on or off the job. For example, videos demonstrated how mindful breathing techniques could be used to manage air consumption for firefighters or how mindful movement and yoga postures can be adapted to required combat skills for law enforcement. These job-specific adaptations may have been useful not only in engaging first responders but also in providing complementary techniques to operational training, which previous studies suggest is more effective than generic stress management training and may even contribute to better psychological outcomes after trauma exposure [70,71]. Second, this type of training would enable first responder organizations to reach their employees, who are often geographically dispersed, using a standardized approach that is convenient and flexible. The web-based format would allow employees to complete the required training with minimal impact on their regular duties and responsibilities. This format also provides employees with access to practical tools and strategies on demand. Although further rigorous evaluation through an RCT is warranted, our results suggest that a web-based mind-body tactical resilience training program for first responders may be a feasible and acceptable means of engaging this population group, who are known to report high rates of stigma and low engagement in psychological interventions [6].

### Limitations and Future Research

Our study has a number of important limitations, most notably the small sample size and high study attrition rate, where only 29% (12/42) of the enrolled participants completed the follow-up assessment. Although high dropout rates for program completion are common for many web-based interventions [72], as previously discussed, the low retention rate for the follow-up assessment may also indicate potential bias caused by nonresponse. The opt-in approach to recruitment may have also introduced some level of selection bias in the sample. Recruitment was facilitated by an external provider of resilience training through their known networks, and it is possible that those who participated in and completed the program were intrinsically motivated to take part in mind-body resilience training. Thus, limited insights were gained into the overall acceptability of the program. There was also a substantial proportion of participants (19/42, 45%) who met the criteria for PTSD at baseline, which is considerably higher than PTSD rates found in most first responder populations [73]. Although, on the one hand, this likely indicates a level of sampling bias, it is unclear if this may also point to the appealing nature of this type of training program for first responders with PTSD symptoms. It is not known if participants had commenced or modified any mental health treatment during the study, which may have influenced the results. However, all participants (42/42, 100%) were active-duty first responders, and none were on long-term sick leave. The lack of effect on mental health outcomes may also be due to the lack of power and small sample size. As our study used a short follow-up period, conclusions about the persistence of any intervention effects over time are unclear because of the lack of extended follow-up assessments. Although this was intended as a feasibility study, the absence of a control group limits any conclusions regarding the effectiveness of the intervention. The use of self-reported data for psychological and process measures was also a limitation; however, these scales are well validated with the exception of psychological preparedness, which was modified specifically for this study. Further research using a more representative sample is necessary before any firm conclusions can be drawn on effectiveness as well as the extent to which this type of training may be effective as a form of prevention or treatment strategy. It is also not yet known what, if any, were the stand-alone effects of each component and how these may have contributed to mental health outcomes. Future research should examine the precise underlying mechanisms that are influenced by the strategies used as part of any mind-body resilience training for first responders. Finally, although our study adopted recommended web-based engagement strategies allowing for interaction with facilitators and peers [35], feedback received from participants suggested that the discussion forums (3/12, 25%) or live Zoom classes (if attended; 1/6, 17%) did not appear useful. It is possible that the lack of anonymity for these interactive components may have discouraged participation among those who preferred to remain anonymous, and scheduling of live classes might have occurred at an inconvenient time. Future research should examine user preferences for such interactive features and whether there may be any difference between those with mental health disorders and those without. Studies should also seek to understand the reasons behind user interest in such web-based programs and whether those who engage with web-based programs may be doing so to avoid in-person interactions.

### Conclusions

Preliminary findings from this study suggest that this tailored web-based mind-body tactical resilience training program appeared to be feasible and acceptable among active-duty first responders. The use of mind-body exercises to integrate both psychological and physical strategies in a web-based format appears to be well received; however, further refinements to the existing program, implementation, and follow-up study procedures may be required to improve program adherence and follow-up assessment response rates. More research is required on the optimal duration and frequency of this type of program to better inform feasibility, as well as on the inclusion of social support via guided and group-based formats and social media. Given the low rates of help seeking among this population [5], there is great potential for this web-based program to address the current gap in preventative resilience training that could integrate both psychological and physical health strategies for active-duty first responders. Further research using a larger, rigorous trial design is warranted to examine the extent to which this type of web-based training may be effective as a form of prevention or treatment strategy for first responders.

## Abbreviations

K6: 6-item Kessler Psychological Distress Scale
PTSD: posttraumatic stress disorder
RCT: randomized controlled trial
REDCap: Research Electronic Data Capture
YFFR: Yoga For First Responders
